# CD14^hi^CD16+ monocytes phagocytose antibody-opsonised *Plasmodium falciparum* infected erythrocytes more efficiently than other monocyte subsets, and require CD16 and complement to do so

**DOI:** 10.1186/s12916-015-0391-7

**Published:** 2015-07-07

**Authors:** Jingling Zhou, Gaoqian Feng, James Beeson, P. Mark Hogarth, Stephen J. Rogerson, Yan Yan, Anthony Jaworowski

**Affiliations:** Centre for Biomedical Research, Burnet Institute, Melbourne, Victoria 3004 Australia; Department of Medicine, University of Melbourne, Melbourne, Victoria 3050 Australia; Department of Chemical and Biomolecular Engineering, University of Melbourne, Melbourne, Victoria 3800 Australia; Department of Infectious Diseases, Monash University, Melbourne, Victoria 3800 Australia; Department of Immunology, Monash University, Melbourne, Victoria 3800 Australia; Department of Microbiology, Monash University, Melbourne, Victoria 3800 Australia

**Keywords:** Malaria, Phagocytosis, Monocyte subsets, Antibodies, Complement, CD16

## Abstract

**Background:**

With more than 600,000 deaths from malaria, mainly of children under five years old and caused by infection with *Plasmodium falciparum,* comes an urgent need for an effective anti-malaria vaccine*.* Limited details on the mechanisms of protective immunity are a barrier to vaccine development. Antibodies play an important role in immunity to malaria and monocytes are key effectors in antibody-mediated protection by phagocytosing antibody-opsonised infected erythrocytes (IE). Eliciting antibodies that enhance phagocytosis of IE is therefore an important potential component of an effective vaccine, requiring robust assays to determine the ability of elicited antibodies to stimulate this *in vivo*. The mechanisms by which monocytes ingest IE and the nature of the monocytes which do so are unknown.

**Methods:**

Purified trophozoite-stage *P. falciparum* IE were stained with ethidium bromide, opsonised with anti-erythrocyte antibodies and incubated with fresh whole blood. Phagocytosis of IE and TNF production by individual monocyte subsets was measured by flow cytometry. Ingestion of IE was confirmed by imaging flow cytometry.

**Results:**

CD14^hi^CD16+ monocytes phagocytosed antibody-opsonised IE and produced TNF more efficiently than CD14^hi^CD16- and CD14^lo^CD16+ monocytes. Blocking experiments showed that Fcγ receptor IIIa (CD16) but not Fcγ receptor IIa (CD32a) or Fcγ receptor I (CD64) was necessary for phagocytosis. CD14^hi^CD16+ monocytes ingested antibody-opsonised IE when peripheral blood mononuclear cells were reconstituted with autologous serum but not heat-inactivated autologous serum. Antibody-opsonised IE were rapidly opsonised with complement component C3 in serum (t_1/2_ = 2-3 minutes) and phagocytosis of antibody-opsonised IE was inhibited in a dose-dependent manner by an inhibitor of C3 activation, compstatin. Compared to other monocyte subsets, CD14^hi^CD16+ monocytes expressed the highest levels of complement receptor 4 (CD11c) and activated complement receptor 3 (CD11b) subunits.

**Conclusions:**

We show a special role for CD14^hi^CD16+ monocytes in phagocytosing opsonised *P. falciparum* IE and production of TNF. While ingestion was mediated by Fcγ receptor IIIa, this receptor was not sufficient to allow phagocytosis; despite opsonisation with antibody, phagocytosis of IE also required complement opsonisation. Assays which measure the ability of vaccines to elicit a protective antibody response to *P. falciparum* should consider their ability to promote phagocytosis and fix complement.

**Electronic supplementary material:**

The online version of this article (doi:10.1186/s12916-015-0391-7) contains supplementary material, which is available to authorized users.

## Background

It is estimated that there are currently more than 200 million malaria infections per year, resulting in more than 600,000 deaths, mainly of children under five years old and caused by infection with *Plasmodium falciparum* [1]. In addition, infection with *P. falciparum* during pregnancy causes maternal malaria which results in increased incidence of pre-term births, low infant birth weight and maternal anaemia causing significant morbidity and mortality [2, 3].

Antibody-mediated effector mechanisms against the blood stages of the parasite’s life cycle are important in protection against clinical malaria disease: in malaria-endemic regions, acquisition of antibodies to blood-stage parasites is associated with protection against death due to severe malaria by five years of age and with protection against clinical malaria by early adulthood [4]. Important targets of protective antibodies are antigens expressed on the surface of infected erythrocytes (IE) [5], and the major target of these antibodies is a surface protein known as PfEMP1 [6]. In addition, acquisition of antibodies to antigens exposed on the surface of IE that adhere and accumulate in the placenta, and express the PfEMP1 variant known as Var2CSA, occurs in a gravidity-dependent manner and is associated with protection against maternal malaria as well as negative outcomes such as anaemia and low birth weight [7–11].

The effector cells most likely to mediate protective effects of antibodies against circulating blood stage parasites are monocytes, which phagocytose IE [12]. They can also accumulate as malaria pigment-laden cells in the placentas of malaria-infected pregnant women [13–15]. Monocytes phagocytose IgG-opsonised IE via Fcγ receptor-mediated mechanisms [16, 17] and secrete both pro-inflammatory and anti-inflammatory cytokines and growth factors in response to parasite ingestion which may aid in both parasite clearance and in limiting inflammation [18, 19]. Circulating human monocytes exist as separate subsets which are identified by their expression of CD14 (the co-receptor for Toll-like receptor 4 (TLR4) recognition of bacterial lipopolysaccharide) and CD16 (FcγRIIIa: a receptor for IgG). The current convention is to define three subsets of human monocytes: classical (CD14^hi^CD16-), non-classical (CD14^lo^CD16+) and intermediate (CD14^hi^CD16+) monocytes [20]. The biological properties of these subsets are governed by differing expression of pattern recognition and chemokine receptors. CD14^hi^CD16- classical monocytes represent the major population in blood, respond strongly to bacterial products via TLR4 and infiltrate into sites of inflammation in response to the chemokine CCL2 [21]. CD14^lo^CD16+ non-classical monocytes may patrol blood vessel walls and respond to viral ligands via TLR7/8. They express high levels of fractalkine receptor (CX3CR1) but migrate in response to multiple chemokines [21]. CD14^hi^CD16+ intermediate monocytes may represent a transitional form of the maturation of classical monocytes into non-classical monocytes and respond strongly to both viral and bacterial ligands [22]. The role of different monocyte subsets in settings of parasite infection is not known.

While it is recognised that a successful vaccine strategy must generate a robust antibody response to blood stage parasites, the desired functional activities required for protective immunity are less clear. Evidence is accumulating that the ability of antibodies to promote opsonic phagocytosis of blood stage parasites is an important component of immunity [23–26]. However, the major cell subsets mediating phagocytosis and the underlying mechanisms are poorly understood. This knowledge may be crucial for the development of highly protective vaccines. Monocyte phagocytosis of blood-stage malaria parasites has been previously studied using peripheral blood mononuclear cells or purified monocytes which miss interactions between serum components, uninfected erythrocytes and the phagocyte, and have not usually considered individual monocyte subset responses. Here we use a whole blood phagocytosis assay [27] to show for the first time that CD14^hi^CD16+ intermediate monocytes have a much greater phagocytic activity towards trophozoite stage malaria parasites than other monocyte subsets. Our results uncover an essential role for Fcγ receptor IIIa (CD16a) and show that complement opsonisation is required for IgG-mediated phagocytic uptake under physiological conditions and contributes to the high activity of intermediate monocytes against IE.

## Methods

### Ethics approval

Blood was obtained by venepuncture with informed consent from healthy volunteers without a history of malaria infection using protocols approved by The Alfred Hospital Research and Ethics Unit. Five serum samples with high IgG reactivity to CS2 IE were pooled from samples collected from pregnant women in an endemic region of Papua New Guinea [28]. All women gave informed written consent and ethics approval was provided by the Medical Research Advisory Committee, PNG).

### Parasite culture and purification

P. *falciparum* laboratory lines CS2 [29] and E8B [30] were grown in human erythrocytes (group O, Rh^+^, Australian Red Cross Blood Service) at 37 °C with 5 % CO_2_ suspended in RPMI-HEPES medium supplemented with 50 μg/ml hypoxanthine, 25 nM NaHCO_3_, 20 μg/ml gentamicin, 5 % heat inactivated pooled human serum and 5 % Albumax. Gelatin enrichment of knob-expressing IE was performed weekly, and IE were synchronised weekly by resuspension of culture pellets in 5 % sorbitol in water to lyse trophozoite and schizont IE. Mature pigmented trophozoite stage IE were enriched by centrifugation over Percoll gradients to >80 % purity as assessed by counting Giemsa-stained thin blood smears by microscopy.

### Infected erythrocyte labeling and opsonisation

IE were opsonised at a concentration of 5 x 10^7^ trophozoites/mL for 30 minutes at room temperature with rabbit anti-human erythrocyte antibodies (Cappel, MP Biomedicals, LLC; Santa Anna, CA, USA) using a sub-agglutinating 1/800 dilution of antibody in phosphate-buffered saline (PBS). In some experiments CS2 IE were opsonised with 20 % human immune serum from a pool of sera prepared from pregnant woman with placental malaria enrolled in the VT cohort in Papua New Guinea [31]. Opsonised cells were washed in fluorescence-activated cell sorting (FACS) wash buffer (PBS, 2 % new born calf serum) and resuspended in PBS (2 x 10^8^/mL) then stained with 10 μg/mL ethidium bromide (EtBr) for 30 minutes at room temperature. Following labeling with EtBr, cells were washed three times with cold FACS wash buffer and used immediately.

### Whole blood phagocytosis assay

A sample of 5 mL whole blood was collected from healthy volunteers into lithium heparin blood collection tubes by venepuncture and analysed within two hours of collection. Aliquots of 50 μL whole blood were placed in polypropylene FACS tubes, then 1 x 10^7^ EtBr-labeled IE were added. This is a ratio of approximately 200 IE per peripheral blood mononuclear cell. Cells were incubated for 30 minutes at 37 °C, or on ice as a control. After phagocytosis, cells were lysed with 3 mL 0.2 % ammonium chloride for five minutes at 22 °C, then washed with 3 mL cold FACS wash buffer. Supernatant was removed and cells were resuspended in 100 μL PBS. Cells were stained with antibodies for 30 minutes on ice, washed, then fixed with 2 % formaldehyde and analysed immediately by flow cytometry using a FACS Canto II flow cytometer (BD Biosciences, San Jose, CA, USA). Monocyte subsets were identified by staining with anti CD14 APC (M5E2, BD Biosciences) and CD16 FITC (3G8, BD Biosciences), and phagocytosis was determined by measuring EtBr fluorescence in the PE channel. Gates were set using samples incubated with unopsonised, EtBr-stained IE at 37 °C. Flow data were analysed using FlowJo (version 8, Tree Star Inc.). For blocking experiments, blood was pre-incubated with the indicated concentrations of blocking antibodies for 30 minutes at 4 °C before the addition of IgG-opsonised IE. The antibodies used were 3G8 (in house (MH): blocking antibody for CD16), Fab fragment of IV.3 (in house (MH): blocking Ab for CD32a), 10.1 (Santa Cruz Biotechnology, Dallas, TX, USA : blocking Ab for CD64), H1-111 (Biolegend, San Diego, CA, USA: blocking antibody for CD11a), Bear-1 (Abcam, Cambridge, UK: blocking antibody for CD11b) and clone 3.9 (Biolegend: blocking antibody for CD11c). The antibody used to measure C3b deposition of RBC was FITC-conjugated goat fraction to human complement C3, (MP Biomedical 0855167). To measure the effect of inhibiting complement activation on phagocytosis, compstatin (R&D Systems, Minneapolis, MN, USA) was added to whole blood, from stock solutions (2 mg/ml) prepared in PBS, to a final concentration of 0–50 μM, and incubated for 15 minutes on ice before addition of iRBC and transfer to 37 °C to measure phagocytosis.

### Monocyte phenotyping

Aliquots of peripheral blood mononuclear cells (PBMC) from malaria-naive healthy individuals recruited in Melbourne were incubated with predetermined saturating concentrations of the relevant antibodies. Monocytes were gated using forward and side scatter and monocyte subsets identified with CD16 PE Cy7 (3G8, BD Biosciences) and CD14 BV510 (M5E2, Biolegend) or APC (M5E2, BD Biosciences). antibodies used were: CD16 PE Cy7 (3G8, BD Biosciences), CD32a (IV.3 biotinylated Fab fragment + streptavidin APC), CD32b (in house (MH), 63X-21 biotinylated whole IgG + streptavidin APC) [32], CD64 PerCP 5.5 (10.1, Biolegend), CD11a Alexa-488 (HI111, Biolegend), CD11b APC (ICRF44, Biolegend), activated CD11b (CBRM1/5-FITC, Biolegend), CD11c V450 (B-ly6, BD Biosciences), CD35 FITC (E11, Biolegend).

### Intracellular TNF measurements

Phagocytosis of infected red blood cells (iRBC) was performed using 100 μL aliquots of whole blood as described above except that iRBC were not labeled with EtBr. A total of 20 μg/mL brefeldin A and 10 μM monensin was added and the cells incubated for four hours at 37 °C, stained with CD14 APC and CD16 PE Cy7 (30 minutes on ice), then permeabilised (Perm/Wash Buffer 1, BD Biosciences). After 10 minutes on ice, cells were stained with αTNF phycoerythrin (PE) (Mab11, BD Biosciences) for 30 minutes, washed and fixed.

### Imaging flow cytometry

IE were stained with PKH26 (Sigma-Aldrich, Castle Hill, NSW, Australia) according to the manufacturer’s instructions. Aliquots of whole blood (50 μL) were incubated with 5 x 10^6^ PKH26-stained CS2-IE for 15 minutes, and processed as above for whole blood phagocytosis except that cells were stained with CD14 Pacific Blue (M5E2, Biolegend) and CD16 PE Cy5 (3G8, BD Biosciences). Samples were acquired using an ImageStream 100 imaging flow cytometer and analysed using IDEAS software.

## Results

### CD14^hi^CD16+ monocytes in whole blood phagocytose IE more efficiently than other monocyte subsets

**Fig. 1:**
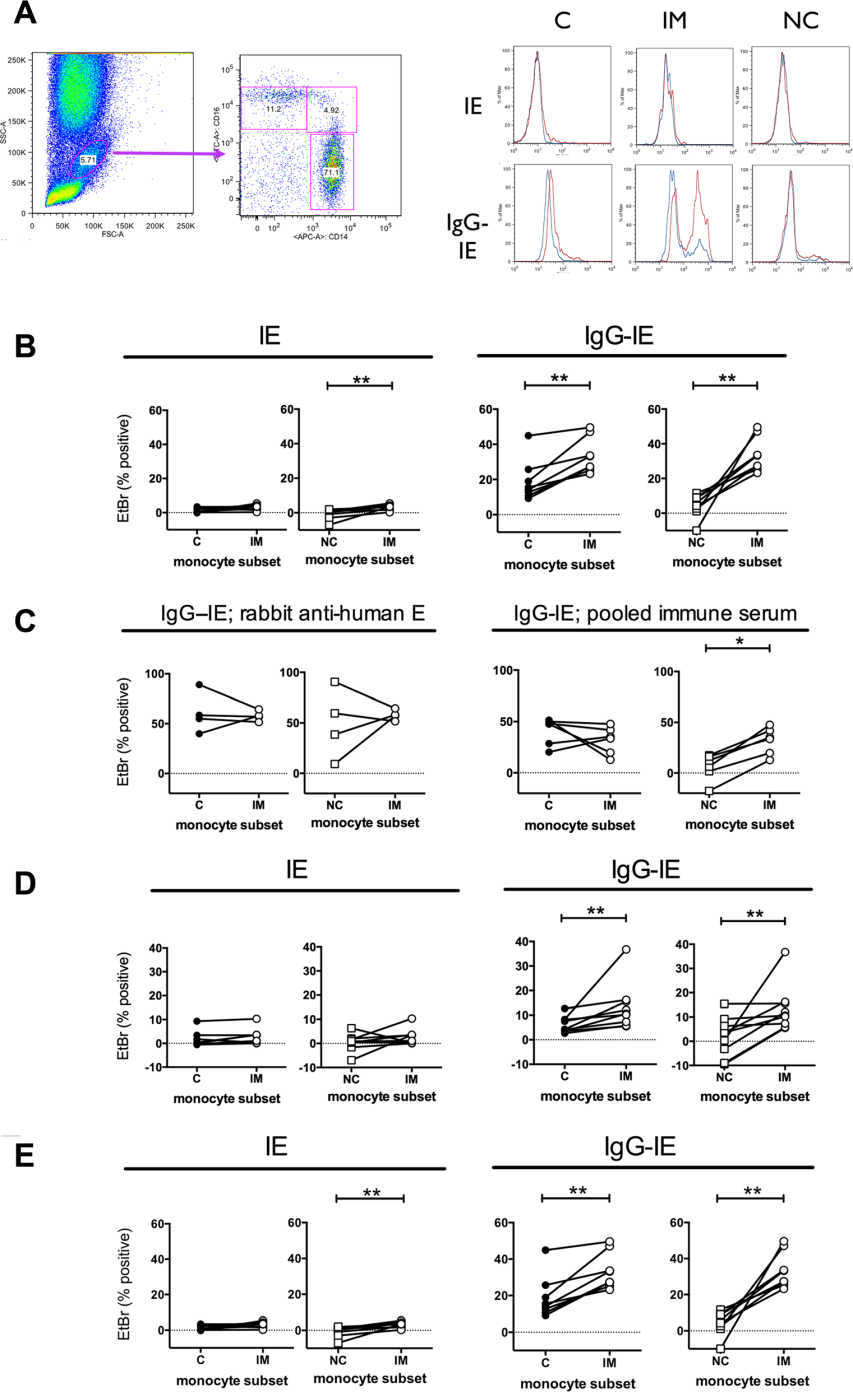
CD14^hi^CD16+ intermediate monocytes phagocytose IE more efficiently than other monocytes. **a** Whole blood was incubated with EtBr-labelled CS2-IE for 30 minutes then uningested RBC removed by hypotonic lysis and washing. Cells were stained with anti-CD14 and CD16, monocytes gated using forward and side scatter then subsets defined as classical (C: CD14^hi^CD16-), intermediate (IM: CD14^hi^CD16+) and non-classical (NC: CD14^lo^CD16+) as shown. Histograms show EtBr staining of the three subsets incubated at 37 °C (red histograms) or 4 °C (blue histograms) with unopsonised (IE, top) or opsonised (IgG-IE, bottom) IE. **b** Phagocytosis using blood from eight separate donors. Whole blood was incubated as in **a** with unopsonised CS2-IE (*left hand panels*; IE) or CS2-IE opsonised with rabbit anti-human RBC antibody (*right hand panels*; IgG-IE) as indicated. **c** Phagocytosis by monocyte subsets of IE opsonised with rabbit anti human RBC was measured using PBMC prepared from four separate donors (*left hand panels*). Phagocytosis of IE opsonised with pooled human immune serum was measured using PBMC prepared from six separate donors (*right hand panels*). **d** Phagocytosis of unopsonised CS2-IE (*left hand panels*; IE) and CS2-IE opsonised with pooled human immune serum (*right hand panels*; IgG-IE) was measured in a whole blood assay as in **a** using blood from nine separate donors. **e** Phagocytosis using blood from six separate donors. Whole blood was incubated as in **a** with unopsonised E8B-IE (*left hand panels*; IE) or E8B-IE opsonised with rabbit anti-human RBC antibody (*right hand panels*; IgG-IE) as indicated. Background phagocytosis measured at 4 °C was subtracted from all data points. The percent phagocytosis by intermediate (IM) monocytes was compared using pairwise comparisons in each case (**b**-**e)** with either that by classical (C) monocytes or non-classical (NC) monocytes, as indicated. Differences between groups were assessed using Wilcoxon matched pairs signed rank test: * p < .05, ** p <0.01. *EtBr* ethidium bromide, *IE* infected erythrocytes, *PBMC* peripheral blood monocytes; *RBC* red blood cells

Whole blood obtained from an individual with no previous history of malaria infection was incubated with EtBr-stained CS2-IE, opsonised or not opsonised with rabbit anti human RBC IgG, and phagocytosis analysed by flow cytometry. The CS2 parasite isolate was chosen both for its relevance to pregnancy-associated malaria and because the lack of CD36 binding potentially minimises the level of non-opsonic phagocytosis. Events within the broad monocyte gate defined by forward and side scatter were analysed on a CD14 versus CD16 dot plot to define the three monocyte subsets CD14^hi^CD16- “classical”, CD14^hi^CD16+ “intermediate” and CD14^lo^CD16+ “non-classical” monocytes (Fig. 1a). The extent of IE phagocytosis by each monocyte subset was determined from the intensity of EtBr fluorescence and compared to the 4 °C negative control. There was little or no phagocytosis of unopsonised IE by any monocyte subset (Fig. 1a, right, upper panels). Opsonisation with IgG increased phagocytosis of IE, particularly by intermediate monocytes (Fig. 1a, right lower panels). Surprisingly, we detected little IE ingestion in the CD14^hi^CD16- or the CD14^lo^CD16+ subsets. CD14^hi^CD16+ monocytes showed much higher phagocytosis of both IgG-opsonised and non-opsonised IE with the CD14^lo^CD16+ monocyte subset showing the least amount of activity (Fig. 1b). These differences were not due to generally higher phagocytic activity of CD14^hi^CD16+ compared to other monocyte subsets since when unopsonised *Escherichia coli* was used as a target the classical subset showed the highest degree of phagocytosis (Additional file 1: Figure S1). Nor was it due to a greater ability of CD14^hi^CD16+ monocytes to phagocytose particles of the size of erythrocytes (7 μm diameter) since when IE were incubated with isolated PBMC instead of whole blood the classical monocyte subset ingested IgG-opsonised IE to a similar extent (Fig. 1c). Since we used rabbit anti-human erythrocyte IgG to opsonise IE to high levels, we next confirmed that CD14^hi^CD16+ monocytes showed enhanced phagocytosis of IE opsonised with human IgG. IE were opsonised with a pool of immune sera from women with placental malaria who had a high titre of antibodies recognising the CS2 isolate; CD14^hi^CD16+ monocytes were again the only subset to substantially phagocytose parasites (Fig. 1d). CS2 is a *P. falciparum* line that expresses Var2CSA. To determine if the increased phagocytic capacity of CD14^hi^CD16+ monocytes was specific to this parasite strain, we incubated whole blood with E8B-IE. E8B is a malaria strain that expresses a mixture of var genes that, in contrast to CS2, promote binding to CD36 and ICAM-1 [33, 34]. CD14^hi^CD16+ monocytes were the only monocyte subset to efficiently ingest IgG-opsonised E8B-IE (Fig. 1e) indicating that the specificity for CD14^hi^CD16+ monocytes is independent of the PfEMP-1 type. More monocytes ingested IE when PBMC preparations were used in the phagocytosis assay compared to whole blood (Fig. 1c c.f 1d). This was true for both CD14^hi^CD16+ monocytes (median phagocytosis = 34.4 c.f. 10.4, p = 0.02) and for CD14^hi^CD16- monocytes (median phagocytosis = 47.9 c.f. 4.22, p = 0.0007).

### The higher phagocytic ability of CD14^hi^CD16+ monocytes, that is evident in whole blood but not PBMC, is not due to lower inhibition by RBC or plasma

Serum and uninfected RBC have been reported to inhibit phagocytosis of iRBC [35]. To test whether uninfected RBC inhibit phagocytosis of IE, and whether this inhibition is less for CD14^hi^CD16+ monocytes, we incubated PBMC with titrated amounts of group O-negative RBC then performed phagocytosis. RBC inhibited phagocytosis by monocytes from all three subsets present in PBMC when added at 25-200x the number of PBMC (Additional file 2: Figure S2A). The maximum ratio of RBC to PBMC used in this experiment was 200:1. This equates to a concentration of 1 x 10^9^/ml which is lower than that found in normal human blood (4-6 x 10^9^/ml). To test whether components present in human plasma inhibit monocyte phagocytosis, PBMC were incubated with varying concentrations of autologous heat-inactivated plasma then phagocytosis measured as above. Plasma inhibited phagocytosis of IgG-opsonised IE by all three monocyte subsets and, in particular, of CD14^hi^CD16+ and CD14^hi^CD16- monocytes equally (Additional file 2: Figure S2B). Thus, uninfected RBC and human plasma both inhibit phagocytosis of iRBC but their presence does not account for the higher phagocytosis by CD14^hi^CD16+ monocytes observed in whole blood.

### Ingestion of IE by CD14^hi^CD16+ monocytes was confirmed using imaging flow cytometry

We next incubated whole blood with opsonised IE for 15 minutes, lysed the uningested erythrocytes, then analysed single cells in focus using imaging flow cytometry (Additional file 3: Figure S3). A shorter time was used to allow ingestion without substantial digestion of the IE. Bright field images of intermediate monocytes confirmed the presence of ingested parasites and the co-localisation of CD16 around the phagosome. Manual counting of ingested parasites using approximately 300 randomly selected bright field images within their respective gates confirmed the higher phagocytic index (PI) of CD14^hi^CD16+ monocytes (33 parasites ingested per 324 monocytes analysed or PI = 10.2 parasites ingested per 100 monocytes) compared to CD14^hi^CD16- monocytes (13/288 or PI = 4.51). The lower extent of phagocytosis in this experiment compared to the experiments depicted in Fig. 1b is due to a lower concentration of IE and the shorter time used.

### Complement opsonisation is absolutely required for phagocytosis of IgG-opsonised IE in whole blood

**Fig. 2:**
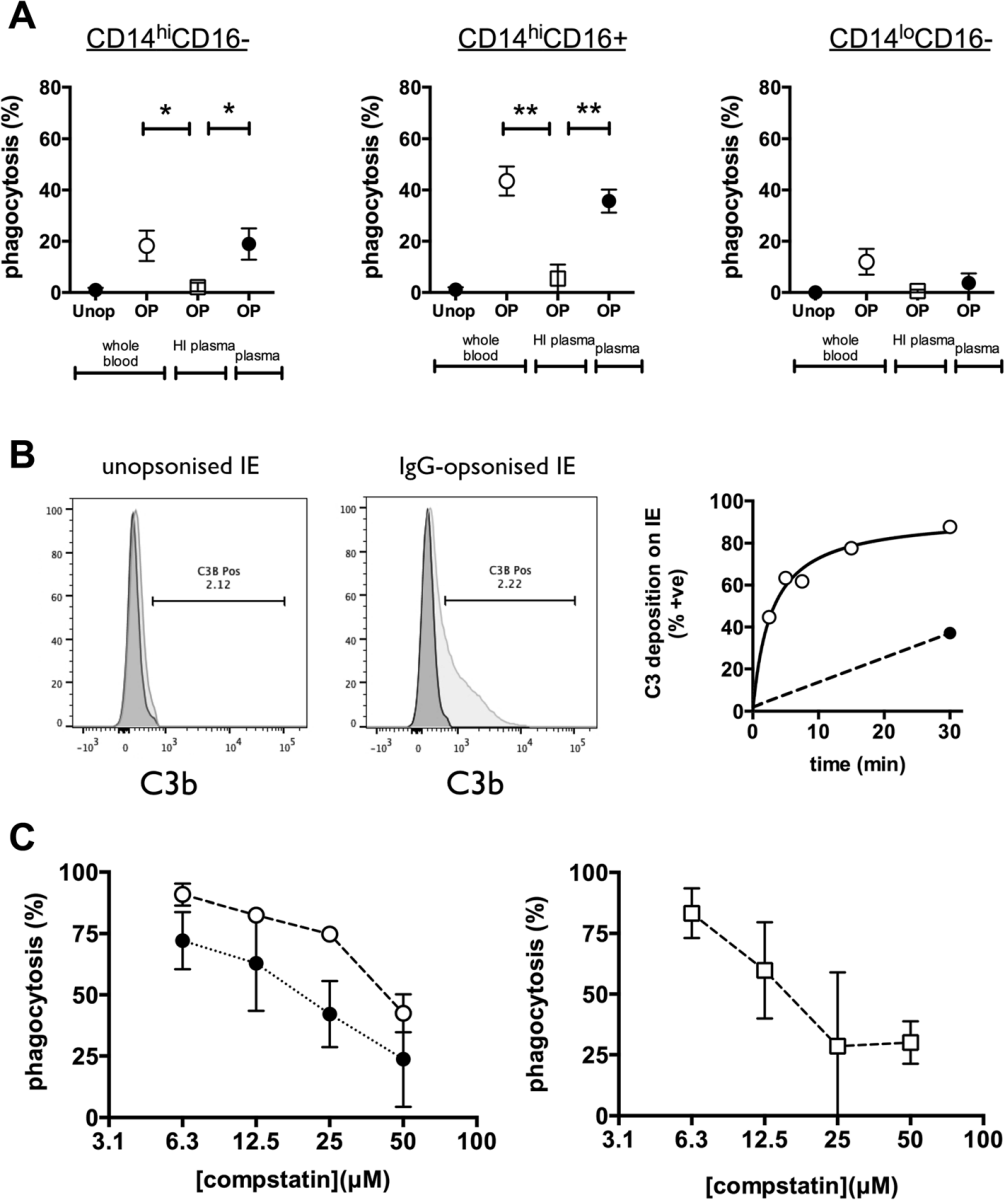
Complement opsonisation is required for monocyte phagocytosis of antibody-opsonised IE **a** Monocyte phagocytosis of CS2-IE (Unop) or CS2-IE opsonised with rabbit anti-human RBC antibody (OP) was determined using whole blood, whole blood reconstituted to its original volume with autologous heat-inactivated plasma (HI plasma) or with autologous plasma (plasma) as indicated. Phagocytosis by classical CD14^hi^CD16- (*left hand panel*), intermediate CD14^hi^CD16+ (*middle panel*) and non-classical CD14^lo^CD16+ (*right hand panel*) monocytes were measured. Data represent mean (sem) of independent experiments using blood from three separate donors. Differences between conditions were assessed by one-way ANOVA using Tukey’s test for multiple comparisons. **b** Unopsonised CS2-IE or CS2-IE opsonised with rabbit anti-human RBC antibody were added to heparinised plasma for 0 and 30 minutes at 37 °C, stained with antiC3, and RBC analysed by flow cytometry. Histograms represent C3 staining at 0 time (dark grey histogram) or 30 minutes (light grey histogram). *Right hand panel* represents C3 staining of CS2-IE opsonised with rabbit anti-human RBC antibody after incubation in heparinised plasma for the indicated times at 4 °C (solid black circles) or 37 °C (open circles). **c** Compstatin (R&D Systems) was added to whole blood from stock solutions dissolved in PBS at the indicated final concentrations then phagocytosis of CS2-IE opsonised with rabbit anti-human RBC antibody by intermediate (open circles) or classical monocytes (solid black circles) determined (*left hand panel*) or non-classical monocytes (open squares) determined (*right hand panel)*. The absolute values of phagocytosis by non-classical monocytes were very low and hence these data are plotted separately. Data represent mean (sem) of independent experiments using blood from three separate donors. *ANOVA* analysis of variance, *IE* infected erythrocytes, *RBC* red blood cells, *sem* standard error of the mean

Since the higher phagocytic activity by intermediate monocytes was observed only when IE were added to whole blood, but not to PBMC preparations, we reasoned that opsonins apart from IgG, such as complement components, might contribute to this activity. We, therefore, centrifuged heparinised whole blood, washed and reconstituted the cells to the original blood volume using either autologous serum or heat-inactivated autologous serum (collected in a separate serum tube at the same time as blood collection), then measured phagocytosis of CS2 IE. Phagocytosis was not affected by washing and reconstituting the blood cells in normal serum, but was abolished when heat-inactivated serum was used (Fig. 2a). These data suggest that complement opsonisation occurs during the 30 minute incubation of IE with whole blood and that this opsonisation is essential for efficient phagocytosis of IgG-opsonised IE by CD14^hi^CD16+ monocytes. To verify this, we added purified, IgG-opsonised CS2-IE to heparinised plasma for various times at 37 °C and measured deposition of C3b onto the opsonised IE by flow cytometry. There was minimal C3b bound to IE at 0 time (Fig. 2b, left and middle panels; dark grey histograms), or after 30 minutes in the absence of IgG opsonisation (Fig. 2b, left panel: light grey histogram), but considerable deposition onto IgG-opsonised IE after 30 minutes (Fig. 2b, middle panel: light grey histogram). C3b was deposited onto IE with a half time of 2.7 minutes (Fig. 2b, right panel). The fact that complement deposition required IgG opsonisation suggests that complement is fixed under these conditions primarily by the classical pathway. To determine the significance of complement to IE phagocytosis by monocytes in whole blood, we next examined the effect of an inhibitor of C3 activation, compstatin. Compstatin inhibited phagocytosis of IgG-opsonised IE by both CD14^hi^CD16- and CD14^hi^CD16+ monocytes (Fig. 2c) showing that even when opsonised with IgG, complement opsonisation is required for efficient phagocytosis of IE. In these experiments phagocytosis by CD14^lo^CD16+ monocytes was very low and was thus excluded from this analysis.

### CD14^hi^CD16+ monocytes produced TNF in response to IgG-opsonised IE

**Fig. 3:**
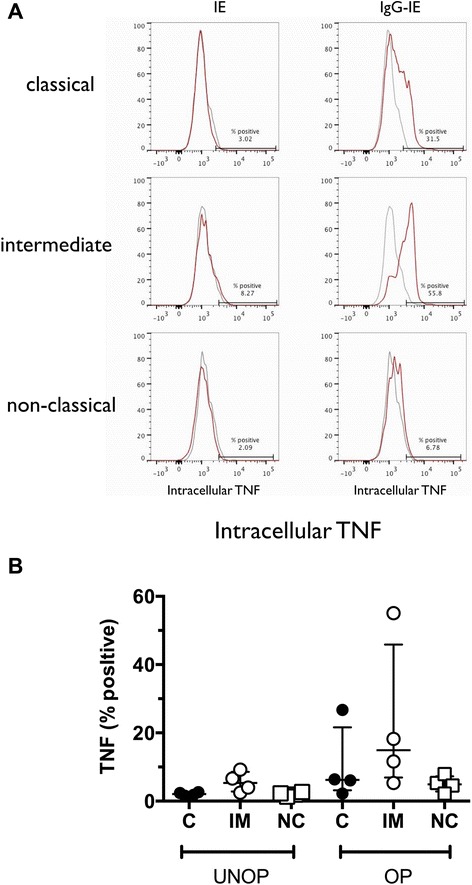
CD14^hi^CD16+ monocytes produce more TNF compared to other monocytes in response to IE. **a** Representative histograms showing intracellular TNF staining of monocytes four hours after addition of CS2-IE (IE, *left hand panels*) or CS2-IE opsonised with rabbit anti-human RBC antibody (IgG-IE, *right hand panels*). Grey histograms: 4 °C controls, red histograms: 37 °C. **b** Median (IQR) of intracellular TNF expression in classical (C; solid black circles), intermediate (IM; open circles) and non-classical (NC; open squares) monocytes from four independent experiments using blood from separate donors. *IE* infected erythrocytes, *IQR* interquartile range

CD14^hi^CD16+ monocytes respond to phagocytosis of bacterial pathogens by producing pro-inflammatory cytokines such as TNF [22]. Since this is thought to be required for both effective immunity to malaria and for immunopathogenesis we determined whether intermediate monocytes produce TNF in response to IE. Whole blood was incubated with unopsonised and opsonised CS2-IE for four hours, then intracellular TNF measured by flow cytometry for all three subsets. There was no TNF production in response to unopsonised IE (Fig. 3a, left hand panels). Both CD14^hi^CD16- and CD14^hi^CD16+ monocytes produced TNF following addition of opsonised parasites, with more of the CD14^hi^CD16+ subset producing TNF in agreement with their greater phagocytic potential, although this difference did not reach significance, whereas CD14^lo^CD16+ monocytes produced very little (Fig. 3a, right hand panels and Fig. 3b).

### Fcγ and complement receptors required for phagocytosis of IE in whole blood

**Fig. 4:**
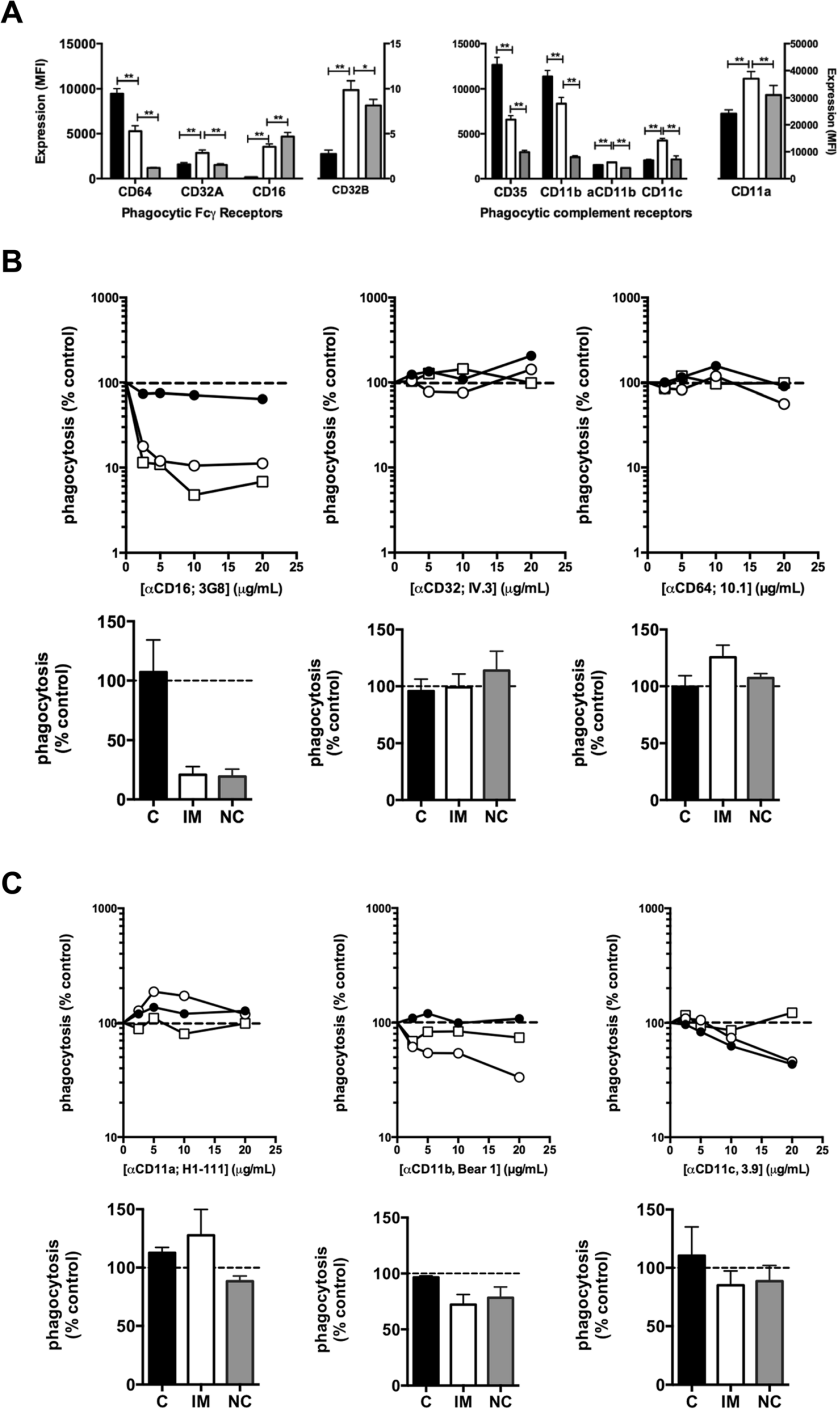
Expression of Fcγ and complement receptors on monocyte subsets and the effect of blocking antibodies on phagocytosis by individual subsets. **a** Expression of Fcγ and complement receptors on monocytes was determined by whole blood staining. Black bars: C, white bars: IM, grey bars: NC. Bars represent mean (sem) of MFI using blood from nine separate donors (eight for CD32b). aCD11b refers to activated CD11b defined by the epitope recognised by the CBRM 1/5 monoclonal antibody. Differences between subsets were assessed using Wilcoxon’s matched pairs signed rank test. * p <0.05, ** p <0.01. **b** Whole blood was incubated for 30 minutes at 4 °C with the indicated concentrations of each blocking antibody before addition of CS2-IE and determination of phagocytosis. Representative dose response curves from four independent experiments, of inhibition of phagocytosis by closed black circles), IM monocytes (open circles) and NC monocytes (open squares) are shown in the *upper panels* and data (mean, sem) from experiments with whole blood from three separate donors conducted using 10 μg/mL of each blocking antibody are shown in the *lower panels*. **c** Effect of blocking LFA-1 (CD11a), CR3 (CD11b) and CR4 (CD11c) on phagocytosis of IgG opsonised CS2-IE. *Upper panels* show dose response of the indicated blocking antibodies and *lower panels* show aggregate data (mean, sem from n =3 independent experiments). Symbols are the same as in **a** and **b**. *C* classical, *IM* intermediate, *N* non-classical

We next phenotyped monocytes from nine independent donors to determine how the subsets differ with respect to expression of receptors involved in binding and phagocytosis of complement and IgG-opsonised targets. Of the phagocytic Fcγ receptors, CD14^hi^CD16+ monocytes expressed significantly higher levels of Fcγ receptor IIa, CD32a, compared to the other subsets (Fig. 4a). The level of the inhibitory Fcγ receptor, CD32b, was also highest in this subset, although this receptor appeared to be expressed at much lower levels than CD32a. With respect to the phagocytic complement receptors, the CD14^hi^CD16+ monocytes expressed the highest levels of the α chain of CR4, CD11c. Of interest, however, was the observation that although CD14^hi^CD16+ monocytes expressed levels of CD11b (the α chain of CR3) that were intermediate between expression on the CD14^hi^CD16- and CD14^lo^CD16+ subsets, they expressed the highest levels of activated CD11b suggesting that inside-out signaling required for CR3 activation was relatively stronger in this subset. CD14^hi^CD16+ monocytes also expressed the highest levels of the α chain (CD11a) of the adhesion molecule LFA-1. Since CD32a is the only Fcγ receptor expressed most highly on CD14^hi^CD16+ monocytes relative to other subsets we reasoned that it may have a unique role in phagocytosis of IgG-opsonised IE. We, therefore, used blocking antibodies to determine which Fcγ receptors are required for phagocytosis. Aliquots of whole blood were pre-incubated for 30 minutes with blocking antibodies specific for CD16, CD32a and CD64, then IgG-opsonised CS2 IE were added and phagocytosis measured after 30 minutes. The blocking antibody specific for CD16, 3G8, inhibited phagocytosis by CD14^hi^CD16+ and CD14^lo^CD16+ monocytes by approximately 90 % at 10–20 μg/mL (Fig. 4b upper panel) but, as expected, had no effect on phagocytosis by CD14^hi^CD16- monocytes which do not express CD16. This inhibition was confirmed using whole blood from three individual donors incubated with 10 μg/mL blocking antibodies (Fig. 4b lower panel). In contrast, blocking antibodies specific for CD32a, IV.3, and for CD64, 10.1, had no effect on phagocytosis by any subset despite these receptors being expressed on all three subsets. Thus, CD16, but not CD32a or CD64 is necessary for phagocytosis of IgG-opsonised IE in whole blood. However, the fact that non-classical monocytes express CD16 but phagocytose IE poorly shows that CD16 expression is not sufficient. Since complement opsonisation was also necessary for phagocytosis of IgG-opsonised IE in whole blood, we investigated the effect of blocking antibodies to the phagocytic complement receptors CR1, CR3 and CR4 as well as antibodies to LFA-1. Antibodies to the α chain of CR3 (CD11b) and CR4 (CD11c) showed minimal inhibition at 10 μg/mL but blocked more efficiently at higher concentrations (Fig. 4c). Anti CD11a did not inhibit phagocytosis by any monocyte subset.

## Discussion

Using a whole blood phagocytosis assay we studied the properties of phagocytes under conditions resembling those *in vivo* as closely as possible. We show that although the CD14^hi^CD16- classical and CD14^hi^CD16+ intermediate monocyte subsets efficiently phagocytose IE in PBMC preparations, only the CD14^hi^CD16+ subset does so in whole blood. While the CD14^hi^CD16+ subset is more efficient at phagocytosis on a per cell basis, the greater number of CD14^hi^CD16- monocytes suggests that they may also phagocytose significant numbers of IE in vivo. We show for the first time that phagocytosis of IgG-opsonised IE required complement opsonisation and was strongly inhibited by inhibitors of complement activation. Antibody blocking experiments showed that in whole blood phagocytosis required expression of the Fcγ receptor CD16, but not CD32a or CD64. Thus, CD16 is necessary but not sufficient for phagocytosis since non-classical monocytes, which also express CD16, failed to phagocytose IE efficiently. This is likely due to their lower expression of CR1, 3 and 4 and lower levels of activated CR3. Complement opsonisation of IE occurred rapidly *in situ* during the assay, primarily via the classical pathway as we did not detect complement deposition on IE in the absence of IgG opsonisation. In whole blood of children with malaria infection, IE have C3b and C4 deposited on their surface [36] but it is not clear whether the limiting factor for efficient phagocytosis is the extent of complement fixation, opsonisation with IgG or both. Our findings show that evaluation of immunity in vaccine trials must also take into account the ability of the elicited antibodies to fix complement as well as to promote phagocytosis. The use of the CS2 strain in this study gives relevance to anti Var2CSA vaccines currently under development for pregnancy-associated malaria and currently funded through to clinical trials. It is essential to understand how antibodies to Var2CSA function in order to design a vaccine with maximum efficacy and to evaluate responses in such trials.

We propose that co-operation between CD16 and complement receptors are required in whole blood for phagocytosis of IgG opsonised IE. Multiple complement receptors are probably involved although a likely partner for CD16 is CR3 (CD11b/CD18) which was more activated on intermediate monocytes than on other monocyte subsets, and partial inhibition of phagocytosis occurred with the blocking antibody Bear-1. CD16 is known to interact with CR3 on monocytes, enhancing its ability to bind iC3b [37]. The selective role of CD16 expressed on monocytes may be due to specific interaction with CR3 and/or signaling differences between it and CD32a. This latter could be caused either by differences in the ITAM motifs located on the FcRγ signaling protein associated with CD16a and that in the cytoplasmic domain of CD32a, or by signaling pathways activated following phosphorylation of the cytoplasmic domain of CD16 [38].

Our data, primarily obtained using IE opsonised with rabbit anti-human RBC antibody, were verified using IE opsonised with human immune serum. Although we did not explore the human IgG isotypes required, studies have shown that anti-malaria antibodies promoting phagocytosis are mainly cytophilic IgG1 and IgG3 [39, 40]. We used two separate laboratory-derived *P. falciparum* lines, CS2 and E8B which were both more efficiently ingested by CD14^hi^CD16+ monocytes. These parasite lines express PfEMP1 adhesion molecules on the surface of the IE that bind with different ligand specificities. Thus, CS2 binds preferentially to chondroitin sulfate A [29] while E8B binds to both CD36 and ICAM-1 [34]. Our observations, therefore, rule out involvement of these receptors in the increased ability of CD14^hi^CD16+ monocytes in phagocytosis.

Imaging flow cytometry verified that under the conditions of our experiments ingestion of IE and not surface binding were measured. This is important since we observed that fragments of bound RBC may remain attached to monocytes following hypotonic lysis of the uningested RBC which can lead to unacceptably high backgrounds when membrane stains are used to label target cells. We found that labelling of the parasite DNA within IE using EtBr was the best approach to use for measurement of IE phagocytosis, but it has the disadvantage that analysis must be performed within 30–60 minutes to avoid loss of the EtBr stain. This lessens the utility of the whole blood assay used here in clinical settings.

Our data have uncovered an important role of CD16 expressed on monocytes in responses to IE. Monocytes express the transmembrane receptor CD16a in contrast to neutrophils, which express the GPI-linked receptor CD16b. Although CD16a and CD16b have nearly identical extracellular domains, they are encoded by separate genes [41]. Polymorphisms of CD32a and CD16b are associated with malaria severity [42–45] that may reflect the ability of splenic macrophages and neutrophils, respectively, to clear opsonised parasites or, in the case of associations with severe anemia, to ingest uninfected RBC. Several polymorphisms in CD16a affect either affinity for cytophilic IgG subclasses [46] or monocyte expression [47]; however, to our knowledge no studies have attempted to associate these or other CD16a polymorphisms with malaria severity or vaccine responses. Given the absolute requirement of IE phagocytosis for CD16 expressed on monocytes revealed herein, such studies are indicated.

The ability of monocytes to phagocytose IE in whole blood is decreased relative to that of PBMC preparations, highlighting the caution that must be employed when interpreting results using PBMC or purified monocytes in phagocytosis assays. This may reflect the presence of inhibitory factors in serum or the large number of uninfected RBC in whole blood. Uninfected red blood cells inhibited ingestion of IgG-opsonised trophozoite stage IE consistent with observations of others using unopsonised schizont-stage IE [35]. We found similar inhibition of all three monocyte subsets, however, suggesting that this is unlikely to underlie their differing phagocytic ability. Interestingly, human RBC membranes contain an inhibitory factor (phagocytosis-inhibitory factor, PIF) which appears to affect CR3 conformation and inhibits ingestion of both C3bi and IgG opsonised latex beads [48]. We also provide evidence that phagocytosis by classical and intermediate monocytes in whole blood is lower than that by these cells in PBMC because of an inhibitory effect of soluble plasma components. It is possible that binding of IgG present in serum to Fcγ receptors may contribute to this, since removal of IgG using protein G sepharose beads reduced inhibition by added plasma (Additional file 4: Figure S4).

Elevated numbers of CD14^hi^ monocytes which co-express the chemokine receptors CCR2 and CX3CR1 have been associated with lower parasitemia and increased ADCI activity in *P. falciparum*-infected individuals with uncomplicated malaria [49]. These cells may define a specific intermediate monocyte population with an important protective role against blood stages of the parasite. It would be of interest to directly compare ADCI activity of different monocyte subsets and the phenotype of monocytes with high phagocytic and high ADCI activity to determine if the same populations are involved. A selective role for CD14^hi^CD16+ monocytes in the response to *P. vivax*-infected reticulocytes has recently been published [49]. This report differs from our study in that increased phagocytosis by CD14^hi^CD16+ monocytes was observed using PBMC preparations incubated for long times (four hours) with purified, infected reticulocytes. In our hands, discrete monocyte subsets cannot be identified after four hours incubation of whole blood with phagocytic targets since CD16 expression on CD16+ monocytes is decreased. A second difference between the two studies is that Antonelli et al. [50] used PBMC prepared from patients with active *P.vivax* infection which were shown to be in an activated state, whereas we have studied responses of individuals with no history of malaria infection and who therefore represent individuals at risk of primary infection. The differences between the two studies may also be due to the different phagocytic targets (*P. falciparum*-infected erythrocytes versus *P. vivax*-infected reticulocytes) used in the two studies. Nevertheless, both studies point to an important role for CD14^hi^CD16+ monocytes in the control and response to blood-stage malaria infection.

In summary, our data show a special role for CD14^hi^CD16+ monocytes in the phagocytosis of trophozoite-stage *P. falciparum* IE. The use of whole blood assays to measure phagocytosis in place of purified PBMC or monocytes reveals that efficient phagocytosis requires both IgG opsonisation and complement components *in situ*. Evaluation of vaccine candidates is increasingly employing functional assays such as phagocytosis assays to determine correlates of immunity. Ideally such assays should use fresh whole blood, although this may not be feasible in some field settings. Our data suggest that care must be taken in interpreting the results of assays using purified cells and that the ability of antibodies to fix complement are likely to be as relevant as opsonic activity.

## Conclusions

In the setting of whole blood, human CD14^hi^CD16+ monocytes are the most efficient subset at ingesting antibody-opsonised IE. This is not observed in assays using isolated PBMC where classical and intermediate monocytes show similar phagocytosis of antibody-opsonised IE. In whole blood, phagocytosis of IgG-opsonised IE requires Fcγ receptor IIIa but not other Fcγ receptors. However Fcγ receptor IIIa is not able to mediate phagocytosis of IgG-opsonised IE on its own but requires complement opsonisation of IE. Assays measuring IE phagocytosis using PBMC or purified monocyte preparations therefore do not detect critical elements of antibody-mediated phagocytosis. We conclude that assays which measure the ability of vaccines to elicit a protective antibody response to *P. falciparum* should consider their ability to promote phagocytosis and fix complement.

## Additional files

Additional file 1:
**Phagocytosis of** ***E.coli*** **by monocyte subsets assayed in whole blood.** Whole blood from a single donor (100 μL) was incubated with 2 x 10^8^ pHrodo® Red-labelled *Escherichia coli* BioParticle conjugates (Life Technologies) for 10 minutes at either 4 °C or 37 °C as indicated, then the cells were stained with CD14-APC and CD16-FITC, and uptake of *E.coli* determined by flow cytometry. The percentage of classical (C; black bars), intermediate (IM; white bars) and non-classical (NC; grey bars) monocytes that had ingested E.coli are plotted. Additional file 2:
**Effect of red blood cells and autologous plasma on phagocytosis of antibody-opsonised IE by individual monocyte subsets.** (A) PBMC were mixed with O- erythrocytes at the indicated cell ratios then phagocytosis of CS2-IE opsonised with rabbit anti human RBC antibody was measured as described in Methods. Percent phagocytosis of IE by each subset is expressed relative to the degree of phagocytosis by the same subset in PBMC alone (zero RBC). Intermediate monocytes (IM), open circles; classical monocytes (C), closed circles; non-classical monocytes (NC), open squares. Data represent mean (sem) of four independent experiments. (B) PBMC washed in PBS containing 2 % FBS were resuspended in PBS containing the indicated final concentrations of autologous plasma, then phagocytosis of CS2-IE opsonised with rabbit anti human RBC antibody was measured as described in Methods. Percent phagocytosis of each subset defined as in (A) is expressed relative to phagocytosis by the same subset in PBMC alone (zero added autologous plasma). Data represent mean (sem) of four independent experiments. Additional file 3:
**AMNIS Image Stream flow cytometric analysis of phagocytosis of antibody-opsonised IE by monocytes.** Phagocytosis of PKH26-labelled CS2-IE opsonised with rabbit anti human RBC antibody by monocyte subsets was analysed by imaging flow cytometry as described in Methods. (A) Gating strategy used to exclude events that were not in focus (*left panel*) or cell doublets (*middle panel*) and to identify the individual monocyte subsets (*right panel*). (B) Representative images of a classical monocyte (C: CD14^hi^CD16-; top panel), an intermediate monocyte (IM: CD14^hi^CD16+; middle panel) and a non-classical monocyte (NC: CD14^lo^CD16+; *lower panel*) from the respective gates defined in (A) above. (C) Representative images of three monocytes within the intermediate monocyte gate containing ingested parasites (indicated on the bright field image by *arrows*) and showing co-localisation of CD16 and PKH26. Due to the differences in brightness of the fluorophores used, it was difficult to depict CD14 on the co-localisation panels; however, all monocytes within the intermediate monocyte gate stained positive for both CD14 and CD16 as shown in the representative cell illustrated in the IM panels in (B). The numbers within the bright field images in Fig. 2c refer to the event number assigned by the IDEAS software. Additional file 4:
**Inhibition of iRBC phagocytosis following addition of heat inactivated serum and Ig-depleted heat-inactivated serum.** A total of 0.5 mL of human serum was heat inactivated for 30 minutes at 56 °C. One half of the serum was depleted of immunoglobulins by incubation with 125 μL Protein G sepharose (Pharmacia) for 20 minutes at room temperature with constant mixing, then the Protein G was removed by centrifugation. PBMC (0.5 x 10^6^) were incubated with 1 x 10^7^ IE (CS2-infected RBC opsonised with rabbit anti-human IgG) in the presence of either 0 or 50 % treated and untreated serum. Phagocytosis by classical (C), intermediate (IM and non-classical (NC) monocyte subsets was measured as described in Methods. The percent phagocytosis of each subset is expressed as a percentage of that of the same subset measured in the absence of added serum (0). Black bars: heat inactivated serum. Open bars; Ig-depleted, heat-inactivated serum.

## Abbreviations

ADCI: antibody-dependent cellular inhibition
C3: complement component 3
C3b: complement component 3b
CR: complement receptor
EtBr: ethidium bromide
FACS: fluorescence activated cell sorting
FBS: foetal bovine serum
iC3b: inactive C3b
ICAM-1: intracellular adhesion molecule-1
IE: infected erythrocytes
iRBC: infected red blood cells
ITAM: immunoreceptor tyrosine-based activation motif
PBMC: peripheral blood mononuclear cells
PBS: phosphate buffered saline
PfEMP-1: plasmodium falciparum erythrocyte membrane protein 1
PI: phagocytic index (number of ingested particles per 100 monocytes)
RBC: red blood cells
TLR: toll-like receptor
TNF: tumour necrosis factor
