# Erratum to: CD14 hi CD16+ monocytes phagocytose antibody-opsonised *Plasmodium falciparum* infected erythrocytes more efficiently than other monocyte subsets, and require CD16 and complement to do so

**DOI:** 10.1186/s12916-015-0530-1

**Published:** 2015-11-30

**Authors:** Jingling Zhou, Gaoqian Feng, James Beeson, P. Mark Hogarth, Stephen J. Rogerson, Yan Yan, Anthony Jaworowski

**Affiliations:** Centre for Biomedical Research, Burnet Institute, Melbourne, 3004 VIC Australia; Department of Medicine, University of Melbourne, Melbourne, 3050 VIC Australia; Department of Chemical and Biomolecular Engineering, University of Melbourne, Melbourne, 3800 VIC Australia; Department of Infectious Diseases, Monash University, Melbourne, 3800 VIC Australia; Department of Immunology, Monash University, Melbourne, 3800 VIC Australia; Department of Microbiology, Monash University, Melbourne, 3800 VIC Australia

## Erratum

It has come to the publisher's attention that the original version of this article [1] contained an error in Fig. 1. Panel 1e was an inadvertent duplication of panel 1b. The correct Fig. 1 has been published in its entirety below.

**Fig. 1:**
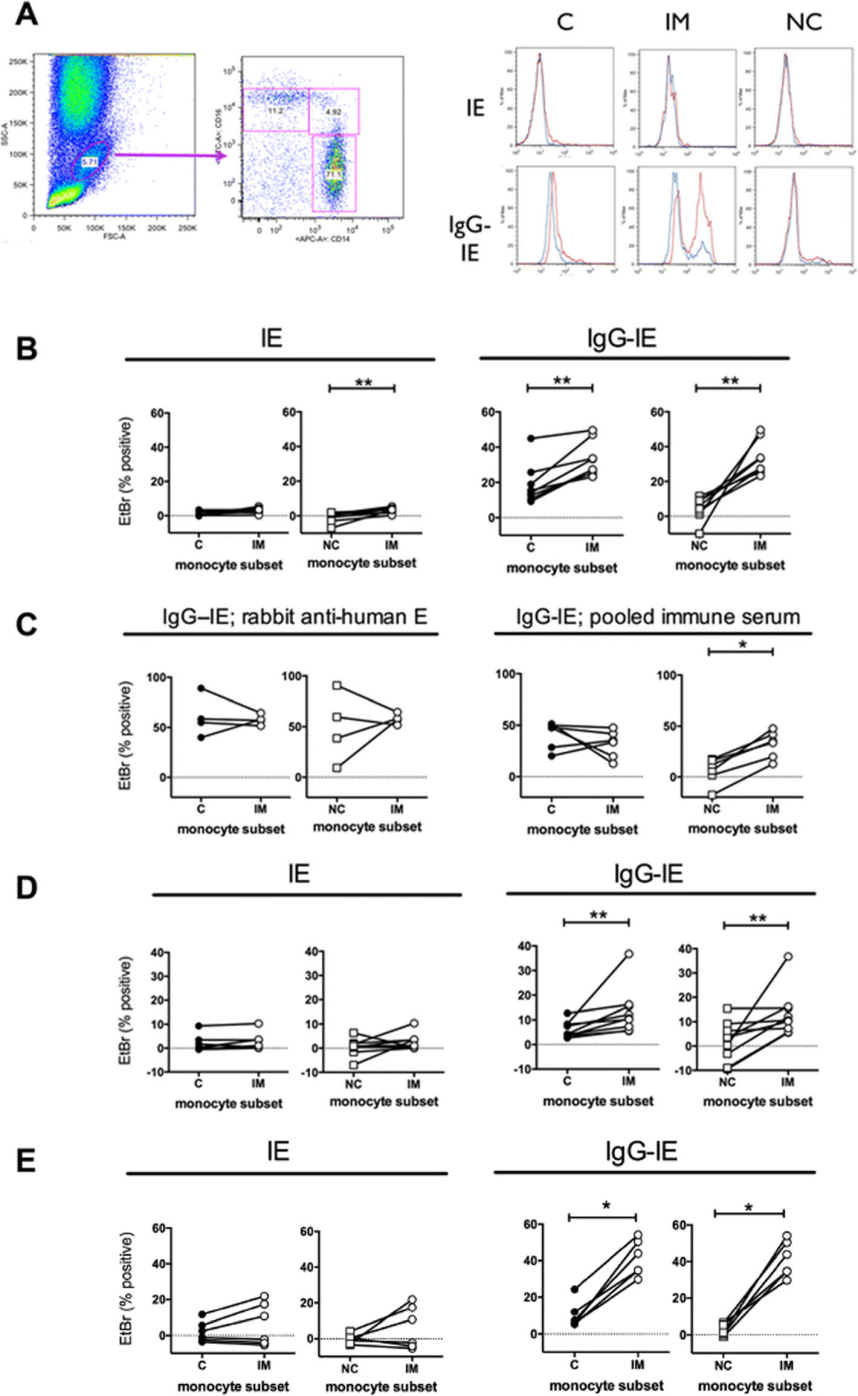
CD14 ^hi^ CD16+ intermediate monocytes phagocytose IE more efficiently than other monocytes. **a** Whole blood was incubated with EtBr-labelled CS2-IE for 30 min then uningested RBC removed by hypotonic lysis and washing. Cells were stained with anti-CD14 and CD16, monocytes gated using forward and side scatter then subsets defined as classical (C: CD14 ^hi^ CD16-), intermediate (IM: CD14 ^hi^ CD16+) and non-classical (NC: CD14 ^lo^ CD16+) as shown. Histograms show EtBr staining of the three subsets incubated at 37 °C (red histograms) or 4 °C (blue histograms) with unopsonised (IE, top) or opsonised (IgG-IE, bottom) IE. **b** Phagocytosis using blood from eight separate donors. Whole blood was incubated as in **a**with unopsonised CS2-IE (*left hand panels*; IE) or CS2-IE opsonised with rabbit anti-human RBC antibody (*right hand panels*; IgG-IE) as indicated. **c** Phagocytosis by monocyte subsets of IE opsonised with rabbit anti human RBC was measured using PBMC prepared from four separate donors (*left hand panels*). Phagocytosis of IE opsonised with pooled human immune serum was measured using PBMC prepared from six separate donors (*right hand panels*). **d** Phagocytosis of unopsonised CS2-IE (*left hand panels*; IE) and CS2-IE opsonised with pooled human immune serum (*right hand panels*; IgG-IE) was measured in a whole blood assay as in **a** using blood from nine separate donors. **e** Phagocytosis using blood from six separate donors. Whole blood was incubated as in **a** with unopsonised E8B-IE (*left hand panels*; IE) or E8B-IE opsonised with rabbit anti-human RBC antibody (*right hand panels*; IgG-IE) as indicated. Background phagocytosis measured at 4 °C was subtracted from all data points. The percent phagocytosis by intermediate (IM) monocytes was compared using pairwise comparisons in each case (**b**-**e**) with either that by classical (C) monocytes or non-classical (NC) monocytes, as indicated. Differences between groups were assessed using Wilcoxon matched pairs signed rank test: * *p* < .05, ** *p* < 0.01. *EtBr*ethidium bromide, *IE* infected erythrocytes, *PBMC* peripheral blood monocytes; *RBC* red blood cells
