# Supplementary figures and images for: CD14hiCD16+ monocytes phagocytose antibody-opsonised Plasmodium falciparum infected erythrocytes more efficiently than other monocyte subsets, and require CD16 and complement to do so
Source: BMC Med. 2015 Jul 7;13:154. doi: 10.1186/s12916-015-0391-7 (PMC4493812; doi:10.1186/s12916-015-0391-7)

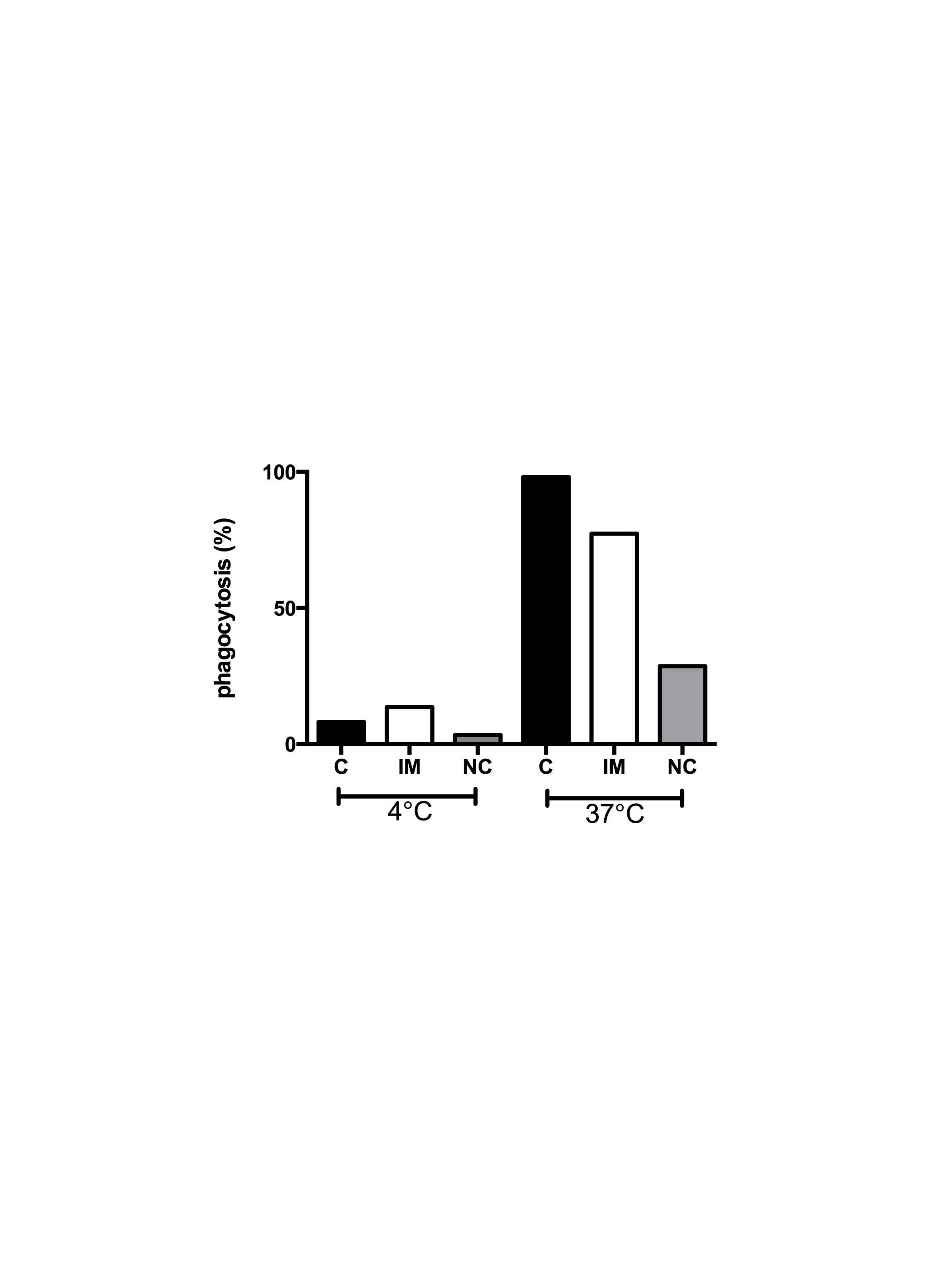

Supplement: Additional file 1: — Phagocytosis of E.coli by monocyte subsets assayed in whole blood. Whole blood from a single donor (100 μL) was incubated with 2 x 10^8^ pHrodo® Red-labelled Escherichia coli BioParticle conjugates (Life Technologies) for 10 minutes at either 4 °C or 37 °C as indicated, then the cells were stained with CD14-APC and CD16-FITC, and uptake of E.coli determined by flow cytometry. The percentage of classical (C; black bars), intermediate (IM; white bars) and non-classical (NC; grey bars) monocytes that had ingested E.coli are plotted. [file 12916_2015_391_MOESM1_ESM.png]

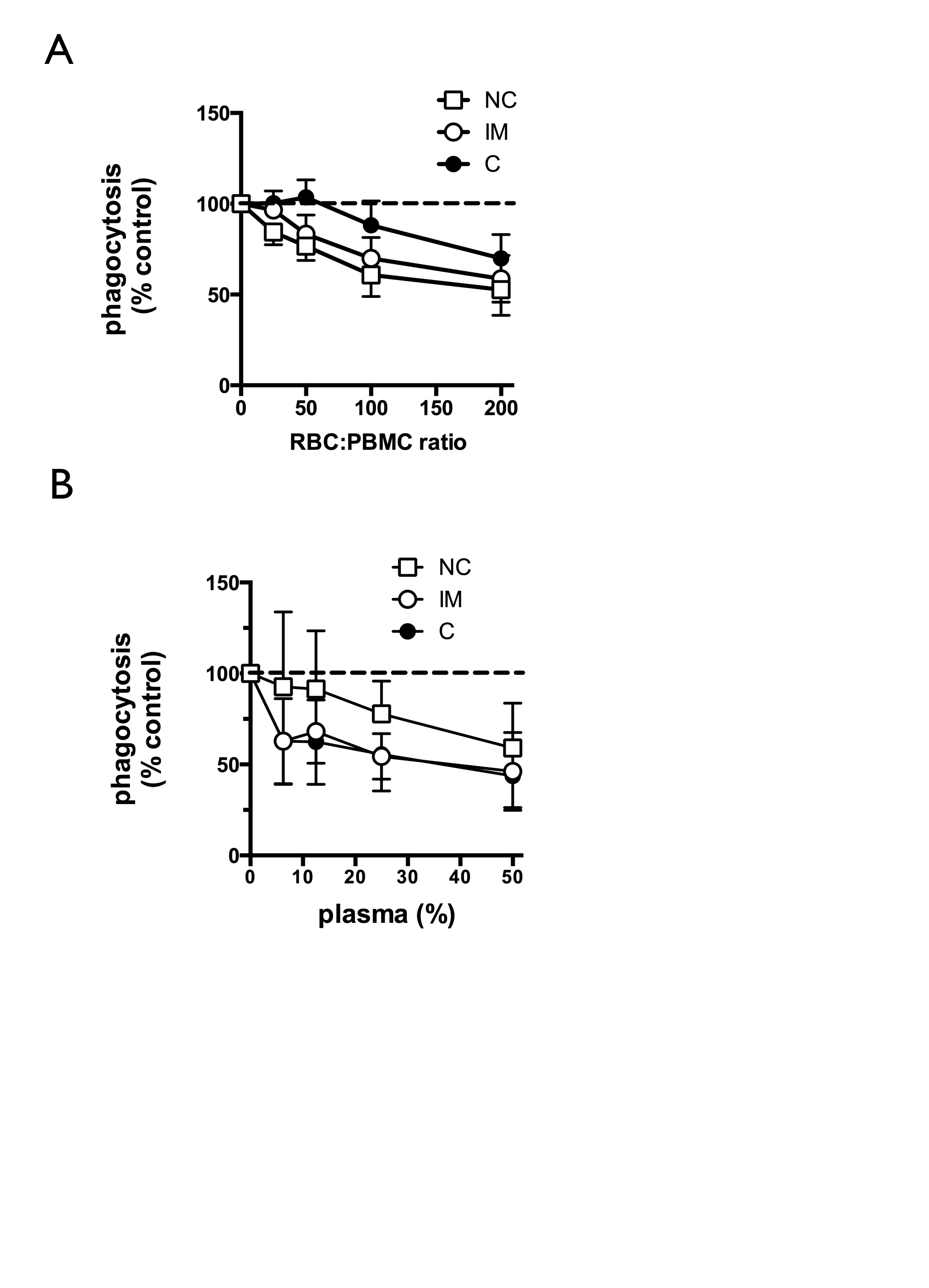

Supplement: Additional file 2: — Effect of red blood cells and autologous plasma on phagocytosis of antibody-opsonised IE by individual monocyte subsets. (A) PBMC were mixed with O- erythrocytes at the indicated cell ratios then phagocytosis of CS2-IE opsonised with rabbit anti human RBC antibody was measured as described in Methods. Percent phagocytosis of IE by each subset is expressed relative to the degree of phagocytosis by the same subset in PBMC alone (zero RBC). Intermediate monocytes (IM), open circles; classical monocytes (C), closed circles; non-classical monocytes (NC), open squares. Data represent mean (sem) of four independent experiments. (B) PBMC washed in PBS containing 2 % FBS were resuspended in PBS containing the indicated final concentrations of autologous plasma, then phagocytosis of CS2-IE opsonised with rabbit anti human RBC antibody was measured as described in Methods. Percent phagocytosis of each subset defined as in (A) is expressed relative to phagocytosis by the same subset in PBMC alone (zero added autologous plasma). Data represent mean (sem) of four independent experiments. [file 12916_2015_391_MOESM2_ESM.png]

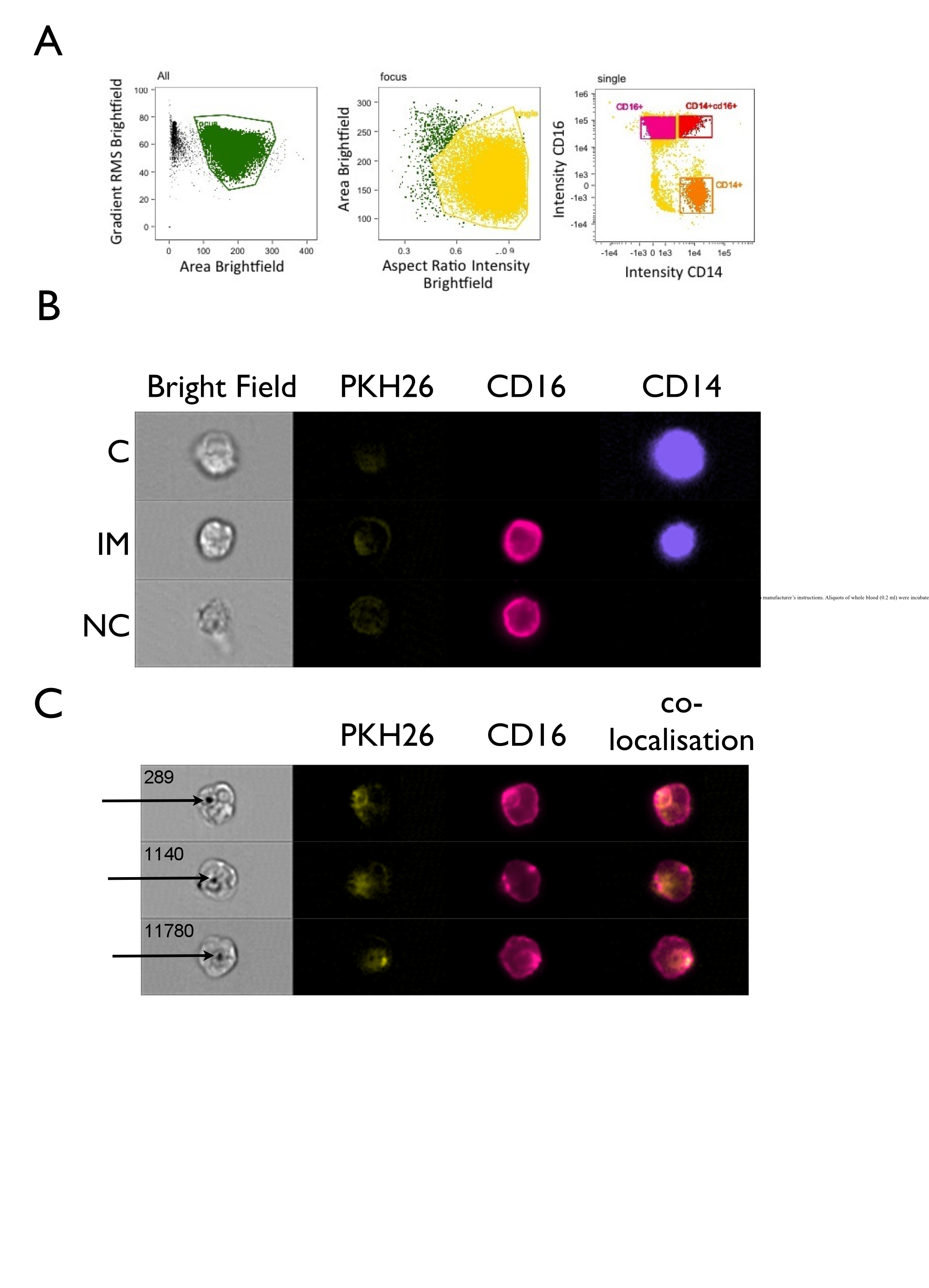

Supplement: Additional file 3: — AMNIS Image Stream flow cytometric analysis of phagocytosis of antibody-opsonised IE by monocytes. Phagocytosis of PKH26-labelled CS2-IE opsonised with rabbit anti human RBC antibody by monocyte subsets was analysed by imaging flow cytometry as described in Methods. (A) Gating strategy used to exclude events that were not in focus (left panel) or cell doublets (middle panel) and to identify the individual monocyte subsets (right panel). (B) Representative images of a classical monocyte (C: CD14hiCD16-; top panel), an intermediate monocyte (IM: CD14hiCD16+; middle panel) and a non-classical monocyte (NC: CD14loCD16+; lower panel) from the respective gates defined in (A) above. (C) Representative images of three monocytes within the intermediate monocyte gate containing ingested parasites (indicated on the bright field image by arrows) and showing co-localisation of CD16 and PKH26. Due to the differences in brightness of the fluorophores used, it was difficult to depict CD14 on the co-localisation panels; however, all monocytes within the intermediate monocyte gate stained positive for both CD14 and CD16 as shown in the representative cell illustrated in the IM panels in (B). The numbers within the bright field images in Fig. 2c refer to the event number assigned by the IDEAS software. [file 12916_2015_391_MOESM3_ESM.png]

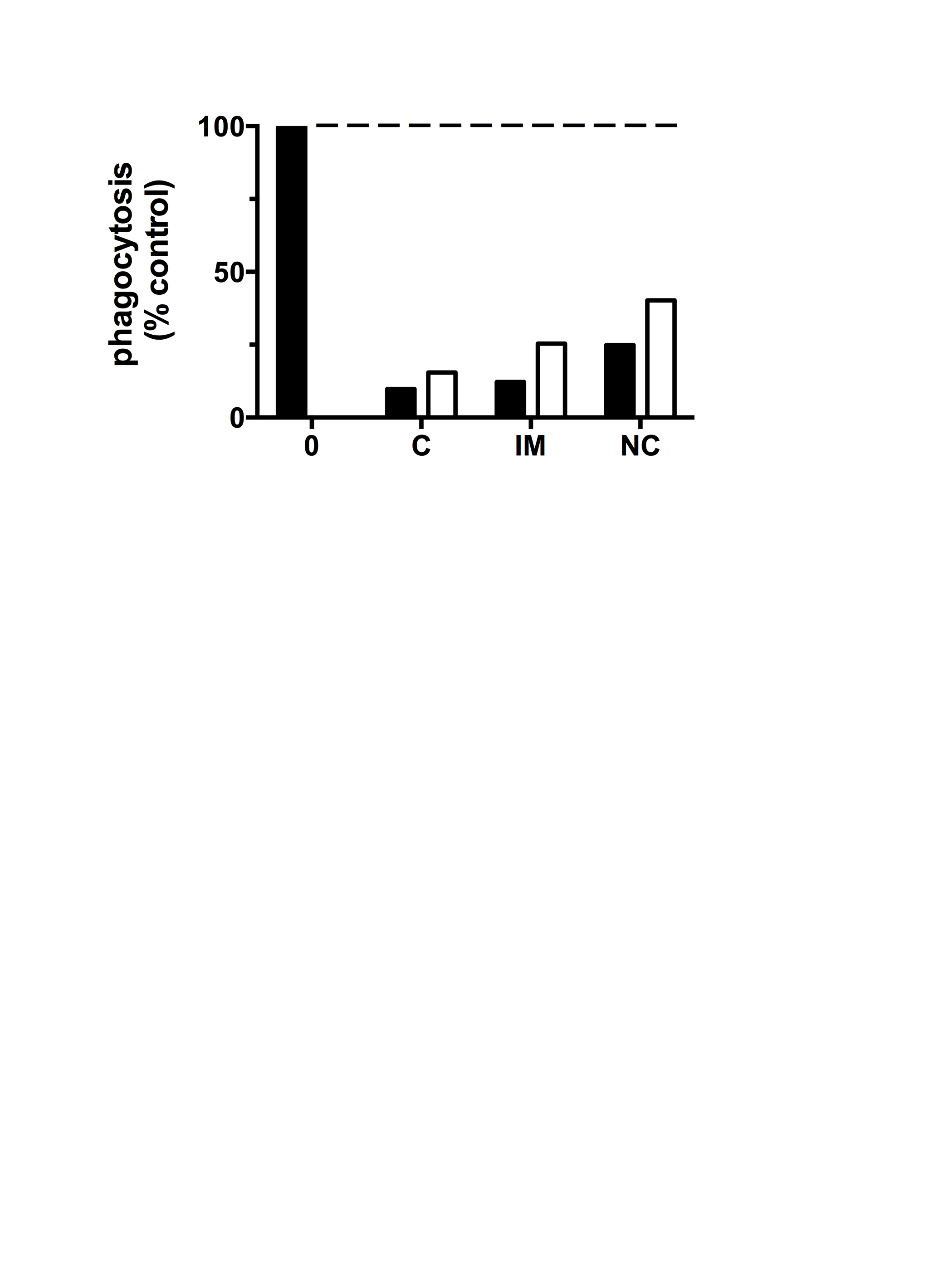

Supplement: Additional file 4: — Inhibition of iRBC phagocytosis following addition of heat inactivated serum and Ig-depleted heat-inactivated serum. A total of 0.5 mL of human serum was heat inactivated for 30 minutes at 56 °C. One half of the serum was depleted of immunoglobulins by incubation with 125 μL Protein G sepharose (Pharmacia) for 20 minutes at room temperature with constant mixing, then the Protein G was removed by centrifugation. PBMC (0.5 x 10^6^) were incubated with 1 x 10^7^ IE (CS2-infected RBC opsonised with rabbit anti-human IgG) in the presence of either 0 or 50 % treated and untreated serum. Phagocytosis by classical (C), intermediate (IM and non-classical (NC) monocyte subsets was measured as described in Methods. The percent phagocytosis of each subset is expressed as a percentage of that of the same subset measured in the absence of added serum (0). Black bars: heat inactivated serum. Open bars; Ig-depleted, heat-inactivated serum. [file 12916_2015_391_MOESM4_ESM.png]
